# The first suicides: a legacy inherited by parasitic protozoans from prokaryote ancestors

**DOI:** 10.1186/1756-3305-6-108

**Published:** 2013-04-18

**Authors:** Emilie Taylor-Brown, Hilary Hurd

**Affiliations:** 1 Centre for Applied Entomology and Parasitology, Institute of Science and Engineering in Medicine, School of Life Sciences, Keele University, Keele, Staffordshire,, ST5 5GB, UK; 2 Centre for the History of Medicine, Faculty of Arts and Humanities, University of Warwick, Coventry, Warwickshire, CV4 7AL, UK

**Keywords:** Parasitic protozoa, Apoptosis, Programmed cell death, Origin of apoptosis, T*rypanosoma*, *Plasmodium*, *Leishmania*

## Abstract

It is more than 25 years since the first report that a protozoan parasite could die by a process resulting in a morphological phenotype akin to apoptosis. Since then these phenotypes have been observed in many unicellular parasites, including trypanosomatids and apicomplexans, and experimental evidence concerning the molecular pathways that are involved is growing. These observations support the view that this form of programmed cell death is an ancient one that predates the evolution of multicellularity. Here we review various hypotheses that attempt to explain the origin of apoptosis, and look for support for these hypotheses amongst the parasitic protists as, with the exception of yeast, most of the work on death mechanisms in unicellular organisms has focussed on them. We examine the role that addiction modules may have played in the original eukaryote cell and the part played by mitochondria in the execution of present day cells, looking for examples from *Leishmania* spp. *Trypanosoma* spp. and *Plasmodium* spp. In addition, the expanding knowledge of proteases, nucleases and other molecules acting in protist execution pathways has enabled comparisons to be made with extant Archaea and bacteria and with biochemical pathways that evolved in metazoans. These comparisons lend support to the original sin hypothesis but also suggest that present-day death pathways may have had multifaceted beginnings.

## Introduction

The word apoptosis was coined by Kerr and colleagues [1] to describe a form of programmed cell death (PCD) previously observed by others. These authors recognised that it was likely to be a general phenomenon seen in all metazoans but, at the time, consideration did not extend to the existence of a similar form of PCD in unicellular eukaryotes, or indeed bacteria and plants [2,3].

The parasitology community has been slow to embrace the notion that parasitic protozoans may, in certain circumstances, self-destruct. Although an apoptosis phenotype was first reported in the trypanosomatids in the mid to late 1990s, it has taken more than two decades for reservations concerning the existence of PCD phenotypes in other lineages to dissipate (e.g. [4,5]). Thinking was undoubtedly coloured by a “top down” approach, generated by our understanding of the function of apoptosis in multicellular eukaryotes as being an essential part of development, growth and resistance to infection. The initiation of a programme for dying does not intuitively appear to contribute to the fitness of an individual unicellular organism and this objection has been a major stumbling block. Additionally, much of the molecular machinery involved in apoptosis pathways in protists has yet to be uncovered, and its resemblance to that of metazoans is only beginning to be recognised. The former issue has recently been explored by several authors [6-9]and will not be discussed in detail in the context of this review.

Despite these concerns, features of apoptosis have now been shown to occur in unicellular members of almost all groups of eukaryotes [10], strongly suggesting that this phenotype is an ancient one that arose when, or even before, the first eukaryotes appeared over 2 billion years ago [11]. If this is the case, we need to resist the temptation to shoehorn the events seen in unicellular organisms into the paradigm of apoptosis as applied to multicellular organisms. Identifying the evolutionary origin of apoptosis may help to resolve this controversy by, for example, explaining the divergence in mechanisms employed by protozoans and metazoans. However, the origin of a trait may not explain its maintenance as it may initially have been selected for one purpose but then maintained, by selection, for another function. Here we are primarily concerned with its evolutionary origin, but will pay brief attention to hypotheses concerning its maintenance.

Plausible hypotheses to explain the evolutionary origins of apoptosis have been around since the turn of the last century, when there was a flurry of interest in this topic [12-17]. Most of these papers focus on the first appearance of the eukaryotic cell and the role that endosymbiosis played in this event [18-20]. We will review these theories and consider whether the latest studies of apoptosis phenotypes and molecular mechanisms in protozoan parasites support any of them.

## Review

### On semantics

The recent boom in literature primarily concerned with cell death mechanisms in unicellular organisms has led to debate over what constitutes PCD and when, if at all, the term apoptosis should be applied to lower eukaryotes. Whilst death itself is relatively easy to define, the process of dying is a much more elusive phenomenon [21]. However, with reference to cell death in higher eukaryotes, the Nomenclature Committee on Cell Death (NCCD) has set out and revised recommendations for the use of terminology to define forms of PCD [22,23]. They do not regard apoptosis as being synonymous with the term PCD, which should be used as a catch-all term to describe organised cell death mechanisms. The term apoptosis is used to describe a specific type of PCD based on morphological criteria including chromatin condensation and nuclear fragmentation [1]. Although these morphological changes are often associated with the loss of mitochondrial membrane potential and the activity of catabolic enzymes such as the caspases, non-mitochondrial and ‘caspase-independent’ pathways also exist [24]. Thus they are not defining features. As it is likely that PCD first evolved in unicellular lineages, and terminology has already been established with respect to cell death in metazoans, application of the same terminology to protozoans will help to clarify links between these death mechanisms. In this respect, PCD should be regarded as an umbrella term that is not precise enough to use when discussing protozoan death pathways that exhibit many of the specific features of metazoan apoptosis [25]. We will use the term apoptosis, rather than apoptosis-like, to refer to this specific form of cell death. This is in line with recent publications by other colleagues in this field [6,25,26].

### Parasitic protozoans exhibiting an apoptosis phenotype

Many studies have now shown that parasitic protozoans, ranging from kinetoplastids to alveolates, exhibit an apoptosis phenotype (reviewed in [25]). Self-destruction pathways involving or by-passing mitochondria have been described, and molecular components are now coming to light, including cysteine proteases [27] apoptosis-related proteins [28] and endonuclease G [29]. In common with multicellular organisms, it is also becoming evident that some parasitic protists have more than one apoptosis pathway.

As an example, the intestinal protozoan parasite, *Blastocystis hominis*, exhibits a complement of ‘death styles’ including both apparent caspase-dependent and independent pathways of apoptosis [30]. Apoptosis features such as cell shrinkage, phosphatidylserine-exposure, apoptotic body formation and cytoplasmic vacuolisation were observed, in conjunction with an increase in caspase-3-like activity. Treatment with the caspase inhibitor Ac-DEVD-CHO resulted in only partial inhibition of DNA fragmentation and did not rescue the cells from PCD, suggesting that a caspase-independent pathway is also present. Although the combined efforts of the pancaspase inhibitor zVAD-fmk and mitochondrial transition pore blocker cyclosporine A completely inhibited DNA fragmentation, cell volume shrinkage and decreased cell counts were still observed, suggesting that PCD pathways were still present [30]. Importantly, the variety of cell death pathways seen in *B. hominis* and other parasitic protozoans, and their differentiation from metazoan mechanisms, need not serve to weaken the argument for shared evolutionary ancestry. The existence of many different means to reach the same end is now recognised as a feature of the self-destruction of metazoan cells as well [24].

### The significance of sharing a bed; apoptosis and the serial endosymbiosis theory

Many authors have proposed that apoptosis evolved from a pathogen/host interaction during the early evolution of the eukaryotic cell [11,13,14,17,31]. The proposal that symbiotic interactions were at the heart of the evolutionary origin of the eukaryotic cell, termed the serial endosymbiosis theory by Taylor [18], is the most commonly accepted explanation for the formation of the eukaryotic cell. It proposes that, following the association between a spirochete–like organism and a thermoplasma, invasion by predatory prokaryotes, termed α-proteobacteria, gave rise to the modern day organelle, the mitochondrion [19]. Margulis [20] suggests that initially the α-proteobacteria were parasitic but, over time, the resolution of conflict between heterogeneous genomes led to enforced cooperation that was beneficial to both symbionts. Most current hypotheses concerning the evolutionary origin of apoptosis are linked to the origin of the mitochondria.

### Are mitochondria the key to the puzzle?

Although apoptosis pathways that do not involve mitochondria are documented, mitochondria are generally of central importance in both the caspase-dependent and caspase-independent pathways. They take up, produce or house molecules required for the initialisation of apoptosis, such as caspases, cytochrome *c* and apoptosis inducing factor (AIF) [32-34]. Kroemer [31] regards mitochondria as the central executioners in apoptosis, acting as death signal integrators. Key to their role in initiating apoptosis is mitochondrial permeability transition (PT), caused by the opening of PT pores. This process allows solutes to diffuse across the inner mitochondrial membrane, thus disrupting the mitochondrial transmembrane potential (ΔΨ_m_), and leading to the subsequent efflux of soluble proteins, such as cytochrome c and AIF. Adenine nucleotide translocase (ANT) is a central component of the mitochondrial PT pore; located in the inner membrane of the mitochondria, its normal physiological role is the exchange of adenine nucleotides across this membrane. ANT, together with cyclophilin D (in the matrix) and the voltage dependent anion channel known as porin, forms the PT pore [35]. Interestingly, mitochondrial porins differ from other eukaryotic porins as they have a β-sheet motif that is amphipathic and they penetrate the lipid bilayer, and form a barrel shaped structure. Other eukaryotic porins have an α-sheet. However, some pathogenic bacteria have porins similar to those in the PT pore, and these can be translocated into a host cell membrane (discussed in [14]). The opening of the pore is triggered by an increase in matrix Ca^+^ concentration and is affected by several effectors including oxidative stress and adenine nucleotide depletion [35]. Kroemer [31] suggests that PT is a universal stress sensor, as many cytoplasmic stressors can cause a conformational change in the PT pore

**Figure 1:**
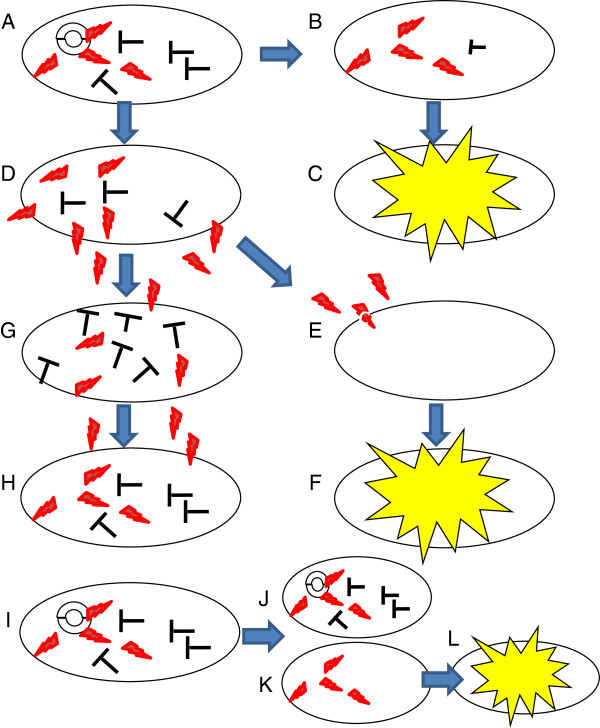
**Addiction modules.** A,B,C: A bacterium containing a plasmid secrets a long-lived toxin and a short lived antidote (**A**). Loss of the plasmid (**B**) results in loss of the antidote before loss of the toxin and this causes cell death (**C**); D,E,F,G,H: A bacterium secreting a toxin and producing an antidote (**D**) will kill a bacterium that does not have the addiction module (**E**) and (**F**) whilst being protected itself (**G**). It is unable to kill a bacterium with the addiction module (**H**); I,J,K,L: A bacterium housing a plasmid with an addiction module divides (**I**) and a daughter cell containing the plasmid survives (**J**) whereas one without the plasmid (**K**) is killed by remaining toxin (**L**); a process known as post-segregational killing.

Many of the components of mitochondria that are involved in initiating an apoptosis pathway were probably present in the proto-mitochondria of the ancestral eukaryote cell. If so, it follows that apoptosis may have evolved as a result of obligatory symbiosis between an α-proteobacteria and its host cell. Indeed some of the underlying genetic machinery may have been acquired from the α-proteobacteria by incorporation into the host genome via horizontal gene transfer (Figure 1). This supposes that apoptosis mechanisms actually have their roots in bacteria and arose as a consequence of the acquisition of aerobic respiration by the eukaryotic cell [14,36]. Below we examine the evidence for this.

### Programmed cell death in bacteria: can we see footprints of the original precursors of apoptosis pathways in extant bacteria?

Hypotheses that propose that ancestral apoptosis pathways were imported into the precursor of the eukaryotic cell by α-proteobacteria gain some support from studies of death pathways in extant bacteria. Several homologues of eukaryotic apoptosis proteins have been identified, including an AP-ATPase domain (found in apoptosis-related proteins such as Apaf −1), a TIR domain, and caspase homologues [37,38], although their involvement in PCD in bacteria has not been ascertained (see Table 1). Molecules that are found in bacterial lineages and also associated with mitochondrial involvement in apoptosis include cytochrome c, components of the PT pore, such as porins and cyclophilins, and structural equivalents of the Bcl-2 family (colicins and diphtheria toxins) (discussed in [31]) (Table 1). Recently Hakensson *et al.*[39] were able to induce features of apoptosis in *Streptococcus pneumoniae* that included calcium-dependent depolarisation of bacterial cell membranes, cell shrinkage, and DNA fragmentation. Downstream degradation pathways involving serine protease and endonuclease activity were also reported. However, proponents of a bacterial origin of eukaryote apoptosis rely heavily on other observations of extant bacteria and their plasmids, in particular, that they produce molecules that cause death, together with inhibitors that block their action; this being reminiscent of the molecular mechanisms for initiation and inhibition of apoptosis seen in metazoans but not, so far, described in parasitic protozoans.

**Table 1:** Eukaryote apoptosis-associated proteins and domain families that have been identified in prokaryotes and parasitic protozoa

**Protein/domain family**	**Archaea**	**Bacteria**	**Parasitic Protozoa**	**Reference**
Caspases/Paracaspases	Haloarchaea Euryarchaeota	*Rhizobium*	**_**	[40,41]
		*Mesorizobium loti*		
Metacaspases	Euryarchaeota	α-proteobacteria	*Leishmania* spp.	[10,26,42-44]
		Cyanobacteria	*Trypanosome* spp.	
		Deltaproteobacteria	*Plasmodium* spp.	
AP-ATPases	*Pyrococcus horikoshii*	α-proteobacteria	_	[17,45]
		Cyanobacteria		[46]
		Actinomycetes		
HtrA-like proteases	_	Many including *Mesorizobium loti*	_	[17]
Endonuclease G	_	_	*Leishmania* spp. *Trypanosoma* spp*.*	[29,47,48]
			*Toxoplasma gondii*	
Apoptosis-inducing factor (AIF)	Many including *Pyrococcus horikoshii*	Many including	**_**	[17,38]
		*Streptomyces coelicolor*		
TIR domain	_	*Streptomyces coelicolor*	**_**	[17]
Cytochrome c	Many including *Aeropyrum pernix*	α-proteobacteria Cyanobacteria Actinomycetes	*Plasmodium spp.*	[49]

Many large plasmids carry a set of genes known as an addiction module. They code for a stable toxin and a labile antidote. For example, type II restriction enzymes and their respective methylases, which form toxin/antidote pairs; the latter affords protection from endonucleolytic attack precipitated by the former. Loss of the plasmid results in bacterial death because the toxin outlives the antidote [2]. The propagation of plasmids expressing toxin/antidote modules is actively upheld as infection becomes coupled with survival; those bacteria that manage to destroy the plasmid will no longer have protection from the toxin, as it is long lasting, and subsequently die. Similarly, daughter cells that did not receive the antidote gene during segregation will die in a process termed ‘post segregational killing’ [50] (Figure 1). Once a plasmid is introduced into the colony, the disadvantage of not having the plasmid is so great that its propagation is actively selected for, with all cured cells facing death - hence the term ‘addiction module’. Originally found in *Escherichia coli,* these addiction modules are thought to maintain the stability of extrachromosomal elements [51,52].

Several of these addiction modules also occur on the chromosomes of bacteria, the most well studied being the *Maz*EF addiction module of *E. coli,* coding for the toxin *Maz*F and the antidote *Maz*E. Stressful conditions such as amino acid or thymidine starvation, UV irradiation, DNA damage and oxidative stress, inhibit the expression of *Maz*E. This leads to the programmed cell death of the majority of the population, with the nutrients released benefiting the survivors [53]. *Maz*EF-like modules have been shown to occur on the chromosomes of many bacteria [51]. Erental and colleagues [53] report that, in *E. coli, Maz*EF-mediated cell death is induced by a quorum-sensing factor (the extracellular death factor) which induces the endoribonucleolytic activity of *Maz*F. Although the *Maz*EF death pathway does not show the major features of apoptosis, these authors also describe an apoptosis-like death pathway in which the membrane is depolarized and DNA is fragmented. Two proteins are involved, RecA and LexA, the latter inhibiting the SOS response to DNA damage. This pathway is only induced by DNA damage, but is inhibited if the *Maz*EF death pathway is operational. Erental *et al.*[53] regard the *Maz*EF pathway as altruistic because it inhibits the apoptosis-like death pathway. However, the molecules involved are not homologous to components of metazoan apoptosis pathways.

A different addiction module, which operates extracellularly in response to nutrient stress, has been described in *Bacillus subtilis.* A *skf* operon directs the production of an extracellular toxin *skf*E, and an antidote *skf*F which confers resistance. The model proposes that a key regulatory protein only operates in a proportion of the population. When activated, cells pump out a toxin that kills any cells in which the regulator is off, thereby providing nutrients for the remaining population, and preventing sporulation of the activated cells [54]. In this and the *Maz*EF system, subsequent survival of some members of the colony in times of stress may contribute to the maintenance of the addiction module within the population.

The concept that ancient bacterial addiction modules may have been the precursors of present day apoptosis pathways will be discussed below.

### From the ‘Wolf at the door’ to ‘Monsters under the bed’

Most theories concerning the evolution of eukaryote cell suicide suggest that it began with an act of parasitism. This parasitism was initially maintained by the α-protobacteria adopting a “mafia-like” strategy to maintain its presence in the host cell by making its removal lethal for the host; acting in essence like an addiction module. The ability to do this may have relied upon the presence of bacterial plasmids operating post-segregational killing mechanisms, as discussed above, or the presence of bacterial toxin/antidote systems originally aimed at killing competitors. These theories are termed addiction hypotheses [11] and are thought by some to explain how α-protobacteria were the precursors of mitochondria-initiated apoptosis pathways.

In 2002, Ameisen [11] discussed alternative scenarios to explain the origin of apoptosis including the proposal that, before the advent of programmed cell death mechanisms, death was only initiated from ‘without’, by toxic environmental conditions or the presence of competitive organisms. The concept of the transfer of ‘death from without’ to ‘death from within’ was discussed by several authors in the late 1990s [13,14,55]. This theory recognises the role of bacterial toxins, originally secreted to kill competitive bacteria, in the emergence of the obligate endosymbiotic interaction that gave rise to the eukaryotic cell. Competing bacteria evolved ‘killer genes’ that encode toxins (antibiotics). These were secreted, killing surrounding bacterial species by inserting a pore into the competing bacterium’s membrane, or damaging its DNA. In parallel, an antidote was produced which provided protection for the initiating bacterium, and indeed any other cell encoding the antidote module. These are addiction modules, as seen in *B. subtilis* (discussed above and see Figure 1). The selection for killer genes that code for toxins, and corresponding antidotes, provided the bacterium with effectors and repressors and conferred a competitive advantage to bacteria containing these modules. The α-proteobacteria/mitochondria precursor may have adapted this mechanism, originally used for killing other competing individuals, to induce the programmed death of their host cell. In other words, the ability to kill competitors may have paved the way for the ability to kill one’s host.

A major advantage may have been gained by the use of an addiction module at this stage in the evolution of the eukaryote cell. A toxin that lysed the host cell may initially have provided the only mechanism for the erstwhile parasite to exit the host cell and transfer to host daughter cells. It may have been initiated when host metabolic condition deteriorated (e.g. lack of nutrients) or replication of the α-proteobacteria leading to density dependent stresses, stimulating the need to find new hosts. Nedelcu and colleagues [56] developed this idea and suggested that the α-proteobacteria originally functioned as a selfish element, increasing the fitness of the invader. This may have led to the production of pro-survival elements (anti-death mechanisms) in the host cell. Stabilization of the selfish element (enforced co-operation) then led to transfer of genes to the host cell and eventually to the present day eukaryotic cell which, in animals, is capable of mediating the death process via the action of pro- and anti-apoptosis elements.

### Beginning with a pore

As already discussed, a prominent feature of apoptosis is a change in mitochondrial ΔΨ_m_ resulting from the opening of PT pores. Components of these pores bear a resemblance to bacterial toxins, such as colicin E1 and streptomycin, which function by inserting pores into the cell membrane [11,14,57]. Kroemer’s hypothetical model [31] proposed that molecules such as porins might have been translocated from the α-proteobacterial membrane to the surrounding phagosome membrane of the host cell, thus facilitating the diffusion of small molecules such as ATP into the host cytosol. It follows that the endosymbiotic bacteria could then sense the ATP levels of the host cell (as a high purine nucleotide concentration keeps the porins closed). If the ATP level dropped, the porins would open, resulting in a flux of Ca^2+^ into the cytoplasm which may have activated caspase-like enzymes that were originally released into the host cytoplasm in an inactive form. Other components such as cytochrome-c may also have been released, causing the ultimate death of the host cell. This would allow the parasitic invader to leave the cell and/or take advantage of the liberated nutrients. Alternatively, the PT pore may have been formed once a mutually beneficial endosymbiotic relationship had been established, with ANT arising later in evolution. Molecules such as Bcl-2 would later evolve (perhaps adapted from alternate functions) to regulate the opening of these pores [14]. Parallel strategies can be seen today in some pathogenic bacteria such as *Neisseria* species (discussed by [57]).

### Molecules from the α-protobacteria

Blackstone and Green [15] proposed an hypothesis for the evolution of apoptosis based on the possession by the parasitic α-protobacteria of an electron transport chain that carried out oxidative phosphorylation. Provision of ATP to its host cell would make this a mutualistic relationship. If host cells were dividing and metabolising rapidly, the α-protobacteria would produce large amounts of ATP, production of reactive oxygen species (ROS) would be low and both symbionts would be advantaged. However, if host cell replication and metabolism slowed, lack of substrate may have reduced oxidative phosphorylation in the α-protobacteria, producing high levels of ROS that may have caused genetic mutations in the host cell. Novel hosts with higher reproductive rates and more rapid metabolism may then have been produced and selected. They hypothesised a role for cytochrome-c, as its release into the cytosol would block the phosphorylation pathway; this and ROS in the cytoplasm would produce a synergistic effect, creating highly reactive mutagenic hydroxyl radicals. Later in the evolution of the eukaryotic cell cytochrome-c could have become a precise signal to alter host phenotype that resulted in cell death, rather than recombination. When Blackstone and Green proposed this hypothesis, the involvement of mitochondria in protozoan apoptosis had not been well recognised and they associated the established role for cytochrome-c signalling with multicellular organisms, excluding nematodes and insects.

### An alternative hypothesis

It is likely that, from the very beginning of cellular life, cell cycle and cell differentiation processes had intrinsic error rates that would have required regulation to prevent cell destruction. Ameisen [11] developed this idea, suggesting that genes associated with these cell processes would have evolved in conjunction with genes encoding for error rate-limiting control mechanisms. He suggested that molecules now involved in apoptosis pathways are as ancient as the origin of the very first cell and proposed that suicide was an unavoidable consequence of self-organisation; a concept he termed the Original Sin Hypothesis. This implies that genetic modules essential to the cell can also be lethal in certain conditions; a form of antagonistic pleiotropy. Thus cell death mechanisms were allegedly adapted over time, congruent with their regulation, not their emergence. This implies that there is no such thing as a *bona fide* cell death programme [11]. Support for this hypothesis is provided by the observation that most genes involved in metazoan cell death programmes can be seen to have other primary functions.

Evidence for this dual-function in life and death is provided by Garrido and Kroemer [58] who report that most pro-apoptosis proteins also have essential metabolic functions distinct from apoptosis. For example, caspase activation is required for cellular processes unrelated to cell death, such as terminal differentiation, proliferation and cytoprotection [59]. Caspase-independent effectors (such as AIF and endonuclease G) are implicated in cellular redox metabolism and mitochondrial biogenesis. Furthermore, cytochrome-c which, when in the mitochondria, is involved in oxidative phosphorylation and ATP production, activates the caspase cascade when in the cytosol [11,21]. Thus key biomolecules such as cytochrome-c comprise a duality and may be switched to cell death function upon receiving a death signal [60]. If this is the case, Ameisen [11] argues, perhaps apoptosis mechanisms evolved in unicellular organisms by adapting already present mechanisms that served ulterior functions.

Kroemer [31] had already pointed out that any central executioner involved in apoptosis must have an alternative function that is vital to the life of the cell, otherwise cells that are completely resistant to apoptosis would evolve. Expanding on this notion, Nedelcu and colleagues [56] also propose that some active cell death mechanisms may have been selected for due to their pleiotropic effects on key cellular processes. Here the loss of cell death machinery would seriously compromise the survival and functioning of the cell, thereby causing a strong selection pressure for pro-survival genes, with apoptosis as its wanton by-product.

If apoptosis was indeed present at the dawn of life, the mechanisms involved appear to have evolved and become more complex. Support for either the addiction model or the original sin model must be sought by investigating the mechanisms underpinning apoptosis in members of ancient lineages that are extant today, such as the parasitic protozoa, to try to understand how their precursors could have given rise to the complex pathways that we see in modern metazoans. In addition, we can examine how and when these mechanisms are deployed in unicellular eukaryotes, in an attempt to understand how suicide pathways could have been maintained before the advent of multicellularity, and why they still exist today.

### Looking for ‘Forbidden Fruit’ on the branches of evolution: the search for apoptosis homologues

Our understanding of molecules involved in apoptosis pathways was initially based on studies of *Caenorhabditis elegans* in the 1990s. Since then homologues of apoptosis regulatory factors have been found in diverse taxa of metazoans, from sponges to mammals [61,62]. Several complex and interlinked pathways have been elucidated, not all reliant upon the activation of caspases or the involvement of mitochondria, suggesting extensive diversification. In mammals, apoptosis occurs by two key pathways, the intrinsic (mitochondrial) and extrinsic (death receptor) pathways. In these pathways two families of proteins, the caspases and Bcl-2 family, are the major protagonists, and degradation of chromatin, causing fragmentation of DNA, is initiated by DNases (reviewed in [62-64]).

The proteins involved in the signalling cascades of apoptosis are complex and contain many domains that allow interaction in diverse pathways. For example, the adaptor domains that connect receptors and effectors (including DD, DED and CARD) whose structure suggests they evolved from a common ancestor [45]. Based on their examination of protein sequences available at the time, Aravidin and colleagues [45] suggested ancient apoptosis machinery was simple, and that domains seen in components of metazoan pathways may have been recruited from proteins initially performing other regulatory functions; an argument that supports the original sin hypothesis. Ameisen [11] pointed out that several components of metazoan apoptosis pathways are probably of ancient bacterial origin, including cytochrome-c, AIF, endonuclease G (EndoG) and Omi/HtrA2 (see Table 1). However there are no bacterial homologues for the genes encoding the Bcl-2 apoptosis-suppression family, although they do share a three dimensional structure with bacterial toxins such as colicin and the diphtheria toxin. It is likely that they were incorporated into apoptosis pathways after the emergence of multicellularity, possibly by horizontal gene transfer.

**Figure 2:**
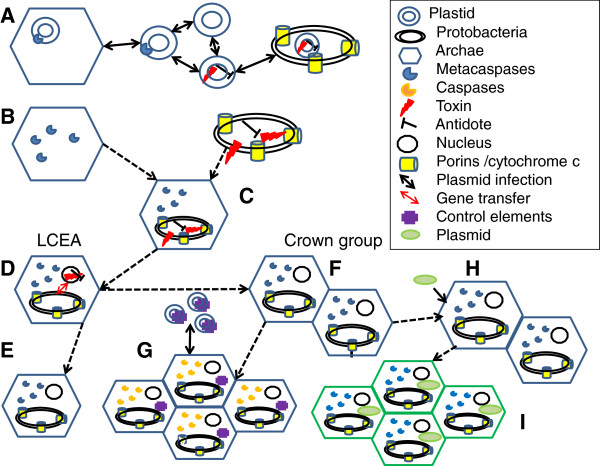
**A hypothetical scenario of the advent of the machinery of apoptosis and its evolution. A**: Initially a prokaryote world containing archaeal cells and α-proteobacteria existed. Multiple gene transfers occurred at this time that may have resulted in incorporation of metacaspases into the archaea and addiction modules into the bacteria. **B**: The ancestral archaeal cell contained metacaspases and the ancestral α-proteobacteria contained an addiction module and elements such as cytochrome c and porins. **C**: The parasitization of the archaeal cell by this α-proteobacteria gave rise to the ancestral eukaryote cell. **D**: A nucleus evolved and gene transfer from the proto-mitochondrion to the host nucleus occurred, giving rise to the last common eukaryote ancestor (LCEA). **E**: The early branching eukaryotes emerged, including the parasitic protozoans. **F**: The last common eukaryote ancestor also gave rise to a crown group exhibiting multicellularity. **G**: The metacaspases family evolved and diversified giving rise to the animals. Caspases and paracaspases and the control elements such as the Bcl family emerged in some groups, possibly as a result of further gene transfer. In general, great diversification of the molecular machinery of apoptosis occurred. **H**: Secondary endosymbiosis occurred between the crown group ancestor and a cyanobacterium. **I**: The stabilization of the plastid gave rise to the plants. This scenario supposes this secondary endosymbiosis occurred after the crown group ancestor became multicellular. However, multicellularity may have arisen twice. There does not appear to be a role in the apoptosis machinery for addiction modules homologous to extant bacteria and these may have been lost once the mitochondrion became stabilized in the host cell.

In addition, homologues of members of the C14 protease super-family, which includes the caspases, have been found in viruses, Archaea, protozoans and bacteria (Table 1). Interestingly, a high number of bacterial homologues were identified in the α-proteobacteria *Bradyrhizobiumjaponicum*, *Haliscomenobacter hydrosis* and *Rhodopseudomonas palustris,* and in the cyanobacterium *Trichodesmium erythraeum*[17]. Koonin and Aravind [17] suggested that eukaryote metacaspases originated from the ancestral α-proteobacteria and that the caspase-paracaspase superfamily may have arisen from a later horizontal gene transfer. Metacaspases were then lost from multiple eukaryotic lineages. However, of particular interest is the recent discovery of caspase-like activity in the haloarchaeon *Haloferax volcanii* in response to salt stress [40]. Caspase-like activity has also been identified in other Haloarchaea; this raises the intriguing possibility that a form of programmed cell death, executed by ancestral members of the paracaspase/metacaspase family, may have already been present in the original archaeon that formed the primary symbiosis with an α-proteobacteria (see Figure 2).

Caspase-like proteases also play a distinct role in the execution of PCD in the cyanobacterium *Trichodesmium*, leading Berman-Frank *et al.*[46] to suggest that these proteases may have been the original executioners. However, despite the evidence that PCD occurs in the cyanobacteria, it is unlikely that the secondary symbiotic event that incorporated the plastid into the ancestral plant group gave rise to plant apoptosis. Plants express all the major hallmarks of apoptosis with evidence of mitochondrial involvement including permeability transition pore formation [65]. But, to the best of our knowledge, plastids play no part in plant PCD pathways.

Further support for the Archaea as a source of molecules involved in apoptosis comes from AIF, which is involved in a caspase-independent pathway. AIF is highly conserved and seen in bacteria and Archaea, but euakryotic AIFs cluster with archaeal orthologues rather than bacterial ones. This suggests that its function in the mitochondria occurred as a secondary event [38].

### How do protozoan parasites fit these paradigms?

The recent publication of sequence data from a variety of protozoan parasites now allows a bioinformatics approach to be taken. This involves examination of lineages of protozoan parasites, already shown to exhibit apoptosis phenotypes, for homologues that support the hypothesis that apoptosis arose before multicellularity. Interrogation of recently annotated genomes of parasitic protists is, indeed, beginning to reveal the presence of domains that are associated with molecules involved in metazoan apoptosis pathways [47] and provides some link with information from prokaryotes.

### Nucleases

Few homologues of molecules involved in mammalian apoptosis pathways have yet been shown to function during apoptosis in these parasites. However, biochemical evidence is coming to light that supports a correlation between nuclease activity and an apoptosis phenotype. Trypanosomatid parasites do not contain orthologues of mammalian caspase-activated DNase (CAD), but putative genes encoding a mitochondrial EndoG have been identified in the genomes of *Leishmania major, Trypanosoma bruce*i and *L. infantum,* and, in each case, endonuclease activity has been demonstrated. Furthermore, there is a mitochondrial targeting signal at the N-terminus of the genes. EndoG has been located in the mitochondria of all three species; apoptosis-inducers cause its release into the cytoplasm, along with cytochrome-c [29,48,66]. Overexpression of EndoG in *Leishmania* spp. led to increased DNA fragmentation (detected using the TUNEL assay) and, in *L. major*, increased sensitivity to H_2_O_2_, and increased spontaneous cell death (*in vitro* and in infected macrophages). Knockdown of EndoG increased resistance to H_2_O_2_[29]. Rico *et al.*[48] also showed that *L. infantum* EndoG is inhibited by concentrations of K^+^ equivalent to that in the intracellular concentration of healthy promastigotes.

Although EndoG is present in yeast and the trypanosomatids, no homologues were found in *Trichomonas vaginalis* or *Plasmodium falciparum*; Kaczanowski and colleagues [47] suggest that it was deleted during the evolution of *Plasmodium* parasites. However their BLAST searches did detect ZEN1 (a key apoptosis DNAse in plants) in the *Plasmodium* genome and they suggest that apoptosis pathways in protozoan parasites may resemble that of plants rather than animals. Interestingly, a ZEN1 homologue is also present in trypanosomatids. Orthologues of the mitochondrial endonuclease AIF were found in the genomes of the apicomplexans *P. falciparum* and *Toxoplasma gondii*, but not in trypanosomatids. Whether these nucleases function in the apoptosis pathways of apicomplexans remains to be determined, but AIF has been shown to be involved in apoptosis in the free-living ciliate, *Tetrahymena thermophila*[67]. Finally, in some amitochondriate organisms, programmed cell death mechanisms have been identified involving granzyme A induced NM23-H1 (a recently discovered DNase) activation [68].

### Proteases

Biochemical evidence for a caspase-like execution pathway has been demonstrated in a variety of protozoan parasites, for example in *B. hominis* and *Plasmodium* spp, although no ‘classical’ caspases have been found in unicellular organisms [42,69-71]. Contenders for the cysteine proteases involved in apoptosis include members of an ancient family of clan CD cysteine proteases - the metacaspases [72-74]. These are present in several parasitic protists [75,76]. The genome of *T. brucei* encodes five metacaspases (MCA1–MCA5) (reviewed in [26]). McLuskey and colleagues [77] have recently determined the crystal structure of MCA2 and they found it to be most structurally homologous to mammalian caspase-7. They state that metacaspase activity is regulated in a different way from caspases, and suggest that caspases and MCA2 evolved independently from a metacaspase-like common ancestor. The recognition that a target for the proteolytic activity of metacaspases, namely Tudor Staphylococcal Nuclease (TSN), is also a substrate for human caspase-3 led Sundstrom *et al.*[78] to suggest that an element of evolutionary conservation exists between apoptosis pathways of trypanosomatids and metazoans. Interestingly, homologues of TSN have been identified across the trypanosomatids and apicomplexa, including *P. falciparum, T. gondi*, *T. cruzi* and *L. major*[47,79].

Caspase-like activity is usually detected by studies using substrate-inhibition assays, however, these assays may not be absolutely specific for the clan CD cysteine proteases that the caspases belong to (discussed in [47]), and the involvement of metacaspases in apoptosis in *P. falciparum* has been contested [80] - although see [42,81]. Other proteases have now been identified as being involved in the apoptosis pathways of parasitic protists. Thus the pan-caspase inhibitor carbobenzoxyl-valyl-alanyl-aspartyl-[*O*-methyl]-fluoromethylketone (Z-VAD-fmk) was not found to inhibit *L. major* metacaspase but it did bind to a cathepsin B-like enzyme that appears to be involved in the apoptosis pathway [82]. This recent finding adds weight to the speculation of Debrabant and colleagues [83] that caspase-dependent and caspase-independent pathways may exist in different *Leishmania* spp. In addition, it has been proposed that a clan CA cysteine protease forms part of the apoptosis molecular pathway *in P. falciparum*[80], and calpain activity has been linked to apoptosis in *E. histolytica*[84]. There is no reason to exclude the possibility that cascades involving more than one clan of cysteine proteases may operate in the execution of apoptosis in some parasitic protozoans

### Other molecules

Ouaissi [85] reviewed work that has characterised members of conserved protein families in *T. cruzi*, including elongation factor 1 (EF-1). One of the subunits, TcEF-1α, was shown to be capable of cytoplasmic-nuclear shuttling during epimastigote growth *in vitro*, and evidence suggests that changes in the level of TcEF-1α may be an important factor in controlling the rate of apoptosis.

An additional protease system resembling the prokaryote ATP-dependent ClpQY machinery has recently been shown to be involved in the *Plasmodium* apoptosis pathway [86]. One system, PfClpQ, is localised in the mitochondria and the other, PfClpP, in the cytoplasm. Disruption of the activity of PfClpY results in loss of mitochondrial ΔΨ_m_**,** activation of VAD-fmk binding proteases and nucleases, and an apoptosis phenotype.

No homologues of the regulatory proapoptosis Bcl-2 family members have been identified in parasitic protozoans. However, Bax was able to induce pore formation and cytochrome c release in *L. major* mitochondria and ectopic expression of Bax caused loss of ΔΨ_m_ and cytochrome c release in *T. brucei*, suggesting functional homologues of this family may yet be discovered (reviewed in [26]). Could these resemble ancient precursors of the Bcl-2 family? Trypanosomatids have also been shown to possess a novel pathway, which operates under conditions of endoplasmic reticulum stress that lead to an accumulation of unfolded proteins. The functioning of this Spliced Leader RNA silencing pathway does not resemble metazoan pathways but results in PS exposure, loss of ΔΨ_m,_ and ROS formation [87].

### A role for mitochondria

The pivotal role of mitochondria in apoptosis pathways is of paramount importance to the potential validity of current theories concerning the origin of cell suicide. The involvement of this organelle in the execution of apoptosis in parasitic protozoa is therefore of particular interest if support for these theories is to be gained from this quarter. The trypanosomatids seem to provide such evidence.

Over a decade ago, a study of *L. donovani* stationary phase promastigotes demonstrated that a decrease in ΔΨ_m_ occurred prior to the induction of caspase-like activity and other features of apoptosis [88]. Antileishmanial drugs have also been shown to initiate an apoptosis phenotype that includes the depolarisation of ΔΨ_m_[88-91], as do other stressors, such as nutrient deprivation and heat stress (reviewed in [26]). Furthermore, an endoplasmic reticulum-induced stress inducer was shown to generate intracellular ROS and the release of Ca^2+^, the elevation of which in the cytosol, caused mitochondrial membrane depolarisation. Interestingly, the reactive oxygen species-dependent release of cytochrome c and EndoG from the mitochondrion, as well as DNA fragmentation and PS exposure, were found to be independent of caspase-like proteins in *L. donovani*[92]; another example of a caspase-independent apoptosis pathway. Mitochondrial superoxide has also been shown to mediate heat-induced apoptosis in *Leishmania infantum*[93].

Exposure to fresh human serum is known to induce apoptosis in *T. cruzi* epimastigotes. Piacenza and colleagues [94] demonstrated that mitochondrial O_2_ radical production and cytochrome c release occurred soon after exposure to human serum. Subsequently, it was confirmed that, here too, the mitochondrion was the key organelle in the signalling process. Mitochondrial Ca^2+^ overload lead to partial dissipation of the inner mitochondrial membrane potential, overproduction and release of superoxide radicals, and cytochrome c release; resulting in an apoptosis phenotype [95].

Loss of ΔΨ_m_ has also been associated with drug-induced apoptosis in erythrocytic stages of *P. falciparum*[71,80,96,97]. However, the situation was found to be more complex when the ookinete stage of *P. berghei* was examined. Over time, an increasing number of ookinetes, developing in cultured gametocytaemic mouse blood, showed loss of ΔΨ_m_ and exhibited markers of apoptosis [98,99]. However, exposure to nitric oxide donors or chloroquine significantly increased the proportion of parasites showing signs of apoptosis, whilst not causing loss of ΔΨ_m_[100,101]. This suggests the existence of alternative pathways that are dependent upon the method of induction of cell death, one of which bypasses the mitochondrion.

### Apoptosis without mitochondria

Despite their lack of mitochondria, the parasitic protozoans *Giardia intestinalis, Entamoeba histolytica*, *Tritrichomonas foetus* and *T. vaginalis* can be induced to exhibit some apoptosis features, such as chromatin condensation, translocation of phosphatidylserine to the outer leaflet of the plasma membrane, DNA fragmentation, and the formation of apoptotic bodies which, in some cases, are inhibited by pancaspase inhibitors [60,84,102]. This also brings into question theories that associate the origin of apoptosis in eukaryotes with the progenitors of mitochondria. However, these and other amitochondriate protozoans do possess organelles, such as hydrogenosomes and mitosomes, which are now regarded as relic mitochondria [68]. Recent reports of seemingly amitochondriate organisms identify these remnant organelles as having double-membranes and also revealed genes encoding for mitochondrially-derived proteins in amitochondrial intestinal parasites [103-106]. This suggests that mitochondria were lost in some branches of the phylogenetic tree, rather than not acquired. In addition, it is hypothesized that hydrogenosomes and mitosomes share a common ancestor with mitochondria and have undergone reductive evolution. The corollary of this is that lateral transfer from mitochondria may offer *one* source for the genetic enigma of modern-day PCD. Previously mentioned non-mitochondrial PCD pathways again suggest multiple systems working in parallel and add more support to the notion that multiple gene infusions, via horizontal gene transfer from bacteria, were fundamental to eukaryote evolution. Hail *et al.*[107] view non-mitochondrial programmed cell death mechanisms as evolutionary back-ups that evolved in tandem, but it is possible that, rather than one system being a back-up for another, different signals induce different pathways.

### Maintenance of apoptosis: conflict or cooperation?

One of the key puzzles concerning the occurrence of apoptosis in unicellular organisms is that of its maintenance. Is this a maladaptive trait that, as discussed above, is an unavoidable consequence of pleiotropy, or is it altruistic? In order to conclude the latter, evidence that other members of the species (probably members of a clonal colony) benefit from the apoptosis of some members must be found. Although protozoan apoptosis has been argued to be an altruistic trait [9,108], evidence of benefit to surviving con-specifics is, so far, scarce. Durand and colleagues [109] have shown that the contents of the unicellular green algae, *Chlamydomonas reinhardtii*, were beneficial to colony members if PCD occurred, but harmful if it was non-PCD. However Segovia *et al.*[110] claim that members of a colony of the chlorophyte *Dunaliella tertiolecta* that were kept in the dark did not gain from death associated with DNA fragmentation, caspase-like activity and morphological features of apoptosis of some members. They regarded it as a maladaptive trait as they assumed that, being obligate photoautotrophs that cannot use dissolved organic compounds, members of the colony could not gain from the death of others. However no tests were conducted to determine whether some colony members could, for instance, benefit from the death of others during short periods of darkness. Convincing evidence does, however, come from *Leishmania*. Disease progression and survival of *L. major* is dependent upon a proportion of the promastigote population undergoing apoptosis [111]. There is also evidence that blood stage *P. falciparum* parasites maintain low parasite density in culture, as a proportion of them undergo apoptosis in response to a form of quorum sensing [97]. The use of apoptosis to control population density in a dividing, clonal population of unicellular parasites would certainly be advantageous if the host were likely to be overwhelmed before parasite transmission occurred. More experimental evidence from a range of parasitic protozoans will serve to determine whether apoptosis is a trait that is maintained because it is beneficial, or is generally a form of antagonistic pleiotropy.

## Conclusion

Evidence gained from the study of protozoan parasites alone suggests that apoptosis originated, not just prior to multicellularity, but also before the divergence of unicellular lineages. There appear to be a few key molecules or domains that are involved in its initiation and execution, though none are common to all organisms investigated thus far. At present, our understanding points to the existence of simple death pathways in lower eukaryotes, with the evolution of additional checks and balances occurring as systems increase in complexity during phylogenesis. Although we have focused on protozoan parasites here, it should be noted that much more is understood of the initiation and execution of apoptosis-like programmed cell death in yeast [112] and studies of free living protists are also contributing to the picture e.g. [113].

As components of these simple apoptosis pathways are being uncovered, it is apparent that they have an essential role to play in the maintenance of the cell, as well as its demise - observations that are supportive of the original sin hypothesis as an explanation for the origin of apoptosis. The key involvement of mitochondria in the initiation of apoptosis in parasitic protozoa supports the hypothesis that “death from within” originated when an ancestral α-proteobacterium infected its first host. However, there is little evidence at present for the existence, in parasitic protozoans, of pro-and anti-apoptosis modules equivalent of those suggested by the addiction hypothesis. Perhaps they are, as yet, undetected because they bear little resemblance to the regulatory molecules of metazoans. Or, maybe, cell suicide did not originate as a consequence of addiction modules.

The primary objective of this review was to focus on the origin of apoptosis, rather than how it might have been maintained in protist lineages. In Figure 2 we suggest a possible scenario for how this occurred. It recognises the major role that horizontal gene transfer is thought to have played in the early evolution of cellular life and the emergence of the ancestral eukaryote cell. The possibility that metacaspase-like proteases were present in the original archaeal cell and that an α-proteobacteria contributed mitochondrial elements, such as the PT pore, is represented. We think addiction modules may have played a role in the stabilization of the relationship between the archaeal cell and the parasitic α-proteobacteria. However, as there is no evidence for modules of this type operating in parasitic protozoa, they may have been lost over time. A major diversification and increase in complexity of the apoptosis molecular machinery occurred after the early branching eukaryotes diverged. This would have included a rapid divergence of the caspase-paracaspase family from the metacaspases [38] and the acquisition of the Bcl-family, possibly by a second infusion of bacterial genes.

Future research into the biochemical pathways involved in apoptosis in parasitic protozoans may shed more light on this issue, but if these life/death modules were indeed imported into the precursor of the eukaryotic cell, it is clear that parasitism was at the heart of the origin of death, as well as of life.

## Competing interests

The authors declare no competing interests.

## Authors’ contributions

HH conceived the idea for this review and both authors contributed equally to its preparation. Both authors read and approved the final version of the manuscript.
